# Using the Timed Up and Go test to measure mobility in non-geriatric patients after pelvic ring injury

**DOI:** 10.1007/s00402-025-05992-9

**Published:** 2025-07-18

**Authors:** Simon Tiziani, Julian Scherer, Patrick Saurenmann, Sasha Halvachizadeh, Roman Pfeifer, Kai Sprengel, Hans-Christoph Pape, Georg Osterhoff

**Affiliations:** 1 University Hospital of Zurich, Zurich, Switzerland; 2 Department of Trauma Surgery and Orthopaedics, BG Klinikum Unfallkrankenhaus Berlin, Berlin, Germany; 3 Chair of Traumatology, Centrum für Muskuloskeletale Chirurgie, Charité-Universitätsmedizin Berlin, Berlin, Germany

**Keywords:** pelvis fracture; outcome; Timed-Up and Go test; validation.

## Abstract

**Introduction:**

Pelvic ring injuries are rare injuries that frequently are associated with prolonged recovery and low return-to-work rates. Since radiological outcome not always correlates with overall patient-reported outcome, the assessement of functional outcome has become a focus of follow up. The Timed-Up and Go test (TUG) would be a simple way to quantify patient mobility, but has not yet been adequately validated in a non-geriatric patient population.

**Methods:**

Consecutive patients younger than 70 years who underwent functional outcome testing as routine follow-up at 6 weeks, 12 weeks, or 6 months after pelvic ring injuries between 11/2017 and 10/2018 were included in this study. In addition to a TUG test, all patients completed a specific functional outcome score for pelvic ring injuries (Majeed-Score) and a general health score (Eq. 5D-3 L).

**Results:**

Forty patients (mean age 40 years, range 18 to 68 years, 24 female), ) of which 28 were treated operatively were included in the study. The mean Majeed-Score was 75.0 (SD 23.3, range 20 to 100) and the mean Eq. 5D-3 L-VAS was 69.9 (SD 22.5, range 5 to 100). The mean time for TUG was 7.8 s. (SD 4.1, range 2 to 22.). TUG time strongly correlated with the Majeed-Score (*r*= − 0.633, *p* = 0.0001) and with the mobility portion of the Majeed-Score (*r*= − 0.524, *p* = 0.001). For a threshold value of 10 s in the TUG test, the ROC analysis revealed a sensitivity of 91% and a specificity of 82% in predicting an impaired Majeed score of below 60 (AUC 0.935).

**Conclusion:**

The TUG correlated strongly with the Majeed score and the mobility part of the Majeed-Score indicating that achieved values reflect the subjective accounts provided by patients. The TUG can be used as a quick screening in non-geriatric patients with pelvic ring injuries to quantify mobility. A TUG longer than 10 s indicates an impaired Majeed score. For academic purposes, however, it should be accompanied by the Majeed-Score.

## Introduction

The incidence of pelvic ring injuries shows two age-related peaks, one in young patients after high-energy trauma such as motor vehicle collisions or a fall from great height, the other in elderly frail patients after low energy trauma like a fall from standing height [1, 2]. Patients with high-energy pelvic trauma injuries are frequently poly-traumatized and the pelvic injury is rarely an isolated one [3–5].

While the mortality rate can be high in the early phase after high-energy pelvic injuries [6], it is only one parameter for assessing the diminished outcome in these patients. Patients who survive the initial treatment have a noticeably reduced functional outcome and low rates of return-to-work [7]. Reporting not only mortality rate and radiographic outcomes, but also the functional outcome has become a standard in pelvic trauma research [8, 9]. Functional outcome scoring usually encompasses region specific scores in combination with general health scores allowing to gauge the influence of the injury on overall functional outcome. The Majeed score is an established and the most frequently used score region-specific score for pelvic ring injuries. Although there exist multiple other specific pelvic scores none of them has been properly validated [10]. The Majeed score combines several dimensions of functional outcome including pain, work-life, sex and mobility [11]. Scoring general health is more heterogeneous with authors choosing numerous systems like the SF-12, SF-36 or a version of the Euroqol e.g. the Eq. 5D-3 L [12, 13]. In addition to these scores, which are predominantly patient-reported outcome questionnaires, specific quantitative tests for e.g. mobility may augment functional outcome scoring.

In case of an elderly study population, the Timed-Up and Go test (TUG) has been well validated as a quantitative test of an individuals’s mobility [14]. The TUG has been developed as a tool to test mobility in frail patients when performing geriatric outcome scoring [15]. It is a simple test that can be conducted in the out-patient clinic or on the ward and which since its inception has been validated for numerous other applications, lately also for pregnant women with pelvic pain [16]. Thus, the aim of this study was to validate the TUG for non-geriatric patients with pelvic ring injuries.

## Methods

### Patients

The study was conducted with approval from the local ethics committee (KEK-2017-01355). All patients gave consent to the use of their health data for research purposes.

Consecutive patients aged 18 to 69 years who were treated for an isolated pelvic injury at our institution between 11/2017 and 10/2018 were included into a prospective database. A Majeed score and Timed-Up and Go Test were performed during routine follow up visits at 6 weeks, 12 weeks, or 6 months and patients were asked to complete a Eq. 5D-3 L questionnaire. Patients without informed consent or with a concomitant injury to the acetabulum, spine or the extremities (*n* = 21) were excluded.

### Scores and tests

The TUG is a test primarily validated as a quantitative measuring tool for mobility in the elderly population [15]. The test was conducted starting with the patients in seating-position. Following the go signal, the patient was asked to stand up, walk three meters, turn around and sit down in the same chair again. The time needed for the exercise, and the use and type of a walking aid were documented. The patient was instructed to complete the task as fast as possible, with less time needed equaling better mobility. In geriatric patients, a time less than 10 s constitutes a normal result [15].

The Majeed score contains seven sections and was completed by the patient under supervision of a surgeon. The scores of all sections are summarized with a higher score indicating a better outcome [11]. Commonly, results > 85 pts. are referred to as “excellent”, 70–84 pts. as “good”, 55–69 pts as “fair” and < 55 Pts as “poor”. For this study, we also analysed a mobility portion of the Majeed-Score (gait unaided, walking distance). Patients who were not sexually active were scored 0 points in the respective section.

The Euroqol 5D-3 L questionnaire is an option to measure the self-reported general health outcome. It encompasses five questions with three escalating answers ranging from items about mobility to fear. Additionally patients can indicate their perceived general health status in form of a visual analog scale ranging from 0 to 100 (Eq. 5D-3 L scale) [12].

### Statistical analysis

Statistical analysis was done by the use of SPSS for windows 25.0 (SPSS, Chicago, Illinois, USA). Data is presented as frequencies and means with range and standard deviation (SD). As the data was not normally distributed, the spearman correlation coefficient was used to assess correlation between the outcome instruments. The level of statistical significance was set at *p* < 0.05.

To determine the threshold value of the TUG that can predict an impaired Majeed score (< 60 points) a receiver operating characteristic curve (ROC) analysis was made with calculation of the area under curve.

## Results

Forty patients (mean age 40 years, range 18 to 68 years, 24 female)) were included in the study. The mean follow-up was 74.6 weeks (SD 95.9, range, 2 to 344) post trauma or surgery.

The predominant fracture patterns were LC I (42%) and LC II (30%) according to Young & Burgess [17] followed by VS (5%), APC I (3%), and LC III (2%) injuries. 18% had isolated fractures of the sacrum without involvement of the anterior pelvic ring. Twenty-eight patients required surgical stabilization.

The mean Majeed score was 75.0/100 pts. (SD 23.3, range, 20 to 100), with a mean of 20.1 out of 24 pts (SD 4.8, range, 6 to 24) in the mobility section of the score. The mean TUG time was 7.8 s (SD 4.1, range, 2 to 22). Only 5% of the patients required a walking aid to complete the test. The mean Eq. 5D-L scale was 69.9 out of 100 (SD 22.5, range, 5 to 100).

**Fig. 1:**
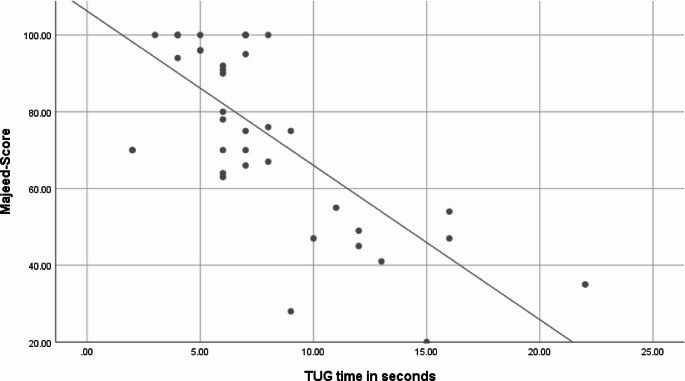
Scatterplot of the TUG test versus the Majeed score

The Majeed-Score and the TUG time showed a strong correlation (*r* = −0.633, *p* = 0.0001, Fig. 1). Similarly, the mobility portion of the Majeed-Score and the TUG time showed a significant inverse correlation (*r* = −0.524, *p* = 0.001).

**Fig. 2:**
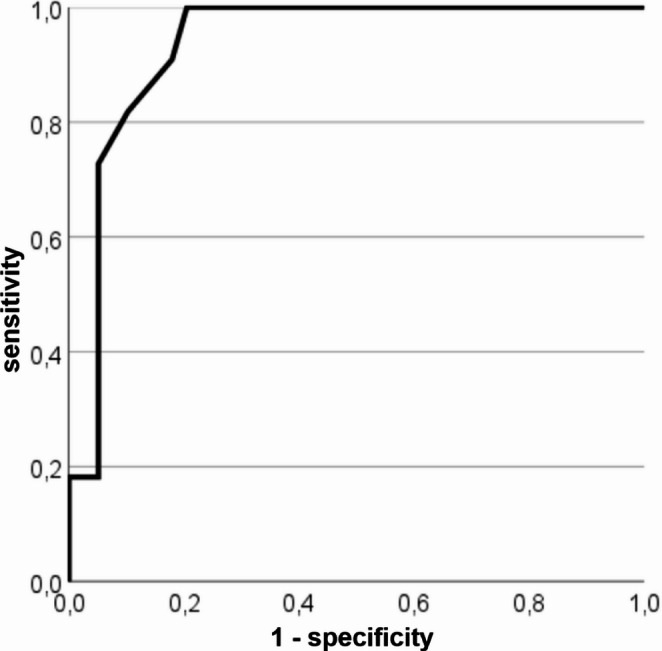
ROC curve for TUG predicting a Majeed score of < 60 points

None of the patients that needed more than 10 s to complete the TUG could reach a Majeed score better than 60 pts. For a threshold value of 10 s in the TUG test, the ROC analysis revealed a sensitivity of 91% and a specificity of 82% in predicting an impaired Majeed score of below 60 (AUC 0.935, Fig. 2).

The use of a walking aid correlated significantly with the mobility portion of the Majeed-score (*r* = −0.385, *p* = 0.014). The Majeed-Score and the Eq. 5D-3 L scale showed a significant positive correlation (*r* = 0.589, *p* = 0.0001). However, the Eq. 5D-3 L scale showed no significant correlation with the TUG time (*r* = −0.216, *p* = 0.182).

## Discussion

The aim of this study was to validate the TUG for use in functional outcome scoring in non-geriatric patients after pelvic ring injury. The results demonstrate a strong correlation between overall Majeed-Score, the mobility aspects of the score and the time needed in the TUG.

Since its inception, the Timed-Up and Go test has seen rising popularity with different groups validating the test beyond its original scope. The test was repeatedly used to score mobility in rheumatoid arthritis [18, 19]. Other groups used the TUG to evaluate risk of falls in patients with multiple sclerosis as well as a general mobility test for this population [20–23]. The test has even been implemented on the very other end of the age spectrum measuring mobility in children with traumatic brain injury [24, 25]. Other studies failed to see the use of the test in evaluating their patient population [26]. Also in the realm of orthopedic surgery, the TUG test has been implemented in patients with lower-limb amputations [27].

We could show that the time needed in the Timed-up and Go Test correlated with the Majeed-Score and its mobility subsections. This seems to indicate that the TUG is an adequate quantitative reflection of the individuals’-answers about mobility that were given in the Majeed questionnaire. None of the patients that needed more than 10 s to complete the TUG could reach a Majeed score better than 60 pts.

The time needed in the TUG did not correlate with the Eq. 5D-3 L scale as a measure of general health. This might indicate that especially in younger patients, a good overall health requires more than just mobility. This seems to be further supported by the significant correlation between the Majeed-Score, adding information about pain, return to work and sexual intercourse, and the Eq. 5D-3 L scale It is known that patients after pelvic trauma rate mental and emotional outcomes as important consequences of their injury [28]. It seems therefore imperative that one avoids reducing overall outcome to a quantitative measurement of mobility.

There are a few limitations to this study. The TUG tests were done as regular follow-up in our out-patient clinic, which means different surgeons supervised the tests. This may introduce the possibility of a bias, even though each examiner was given a detailed introduction into the test. A second limitation is that this study did not look at consecutive scoring of the same patient with the same follow-up instruments. Further studies should try to validate the TUG for different specific follow-up time points.

Additionally, we did not perform a subgroup analysis comparing operative and conservative treatment, as the small number of conservatively treated patients would not allow for a statistically sound comparison and such an analysis was beyond the primary aim of validating the TUG test in this patient population.

In this study, the TUG, Majeed Score, and Eq. 5D-3 L were performed during routine follow-up visits, at 6 weeks, 12 weeks, or 6 months post-injury or surgery. The purpose of the study was to validate the TUG test across various time points rather than at a single fixed follow-up. Therefore, we analyzed all measurements together to reflect its applicability in routine follow-up regardless of the exact time point. A limitation of this approach is that combining measurements from different follow-up time points may mask potential time-dependent differences in the correlation between the TUG and other outcome measures; future studies should consider analyzing these correlations longitudinally at defined intervals.

## Conclusion

The Timed-up and Go Test correlated strongly with the Majeed score and with the isolated mobility part of the Majeed-Score indicating that achieved values reflect the subjective accounts provided by patients. The missing correlation with the general health score Eq. 5D-3 L indicates that not only good mobility is crucial for a favorable overall outcome. This is supported by the fact that the Majeed-Score, which encompasses different aspects of the outcome (pain, work, sex etc.), and the Eq. 5D-3 L correlated positively in this population. The TUG can be used as a quick screening in non-geriatric patients with pelvic ring injuries to quantify mobility. A TUG longer than 10 s indicates an impaired Majeed score. For academic purposes, however, it should be accompanied by the Majeed-Score in order to assess outcome beyond just mobility.

## Data Availability

The data that support the findings of this study are not openly available due to reasons of sensitivity and are available from the corresponding author upon reasonable request. Data are located in controlled access data storage at University Hospital Zurich.
